# Nurse-delivered acupressure on early postoperative gastrointestinal function among patients undergoing colorectal cancer surgery

**DOI:** 10.1016/j.apjon.2023.100229

**Published:** 2023-04-10

**Authors:** Yunhong Liu, Carmen W.H. Chan, Ka Ming Chow, Binbin Zhang, Xue Zhang, Chao Wang, Guangzhong Du

**Affiliations:** a Nursing Department, Qilu Hospital of Shandong University, Jinan, China; b The Nethersole School of Nursing, Faculty of Medicine, The Chinese University of Hong Kong, Shatin, Hong Kong SAR, China; c Department of Acupuncture and Tuina, Qilu Hospital of Shandong University, Jinan, China

**Keywords:** Colorectal cancer, Acupressure, Gastrointestinal function, Randomized controlled trial

## Abstract

**Objective:**

Postoperative gastrointestinal dysfunction occurred up to 25% of patients who undergo colorectal cancer surgery, which could cause severe complications and increase economic burden. This study aims to evaluate the effectiveness of nurse-delivered acupressure on early postoperative gastrointestinal function among patients undergoing colorectal cancer surgery.

**Methods:**

A total of 112 adult patients (≥ 18 years) scheduled to receive colorectal cancer surgery were randomized into two groups. Acupressure was practiced at ST36 for five days after operation, while the control group used gently rubbing skin. Primary outcomes were the time to first passage of flatus and defecation, while the secondary outcomes were the degree of abdominal distention and bowel motility. The Student's *t*-test and Mann–Whitney *U* test or Chi-square test and regression analyses were used, while for repeated measures of outcomes, area under the curve (AUC) was compared between groups and subgroups.

**Results:**

After adjusting for potential confounding variables, acupressure significantly shortened the time to have first flatus passage by 11.08 ​h (95%CI: −19.36 to −2.81; *P* ​< ​0.01). The first passage time of defecation (mean, 77.00 ​± ​36.27 ​h vs. 80.08 ​± ​28.88 ​h), abdominal distention (AUC, 5.68 ​± ​5.24 vs. 5.92 ​± ​4.03), and bowel motility (AUC, 12.09 ​± ​4.70 vs. 11.51 ​± ​3.00) in the intervention group had some improvement although the differences were not statistically significant (*P* ​> ​0.05).

**Conclusions:**

This study indicated that acupressure done by trained nurses could be an effective and feasible solution to promote early gastrointestinal function recovery among patients undergoing colorectal cancer surgery.

**Trial registration:**

Chinese Clinical Trial Registry (ChiCTR-IOR-17012460).

## Introduction

Colorectal cancer (CRC), as a second leading cause of cancer death worldwide in 2020, has imposed considerable harms to human health and well-being with 1.93 million new cases worldwide in 2020.^1^ According to the latest report from China Cancer Centre, the incidence of CRC in China has risen from fifth to second place in the malignant tumor category in the past few decades, with estimated 408,000 new CRC cases in 2016.^2^ The majority of patients with early stage CRC received surgical treatment (84% for colon cancer and 61% for rectal cancer).^3^, ^4^, ^5^, ^6^, ^7^ Upon diagnosis of CRC, surgery is usually the most effective treatment, which can increase survival and reduce morbidity.^8^ Despite this, the development of postoperative ileus has delayed the recovery of patients after CRC surgery.

Postoperative ileus, which is characterized by postoperative nausea and vomiting, bowel distention, lack of bowel sounds, delayed oral feeding, and delayed passage of flatus and stool, is particularly common (up to 25% of patients with colectomy) among patients after CRC surgery.^9^ Postoperative ileus could cause malnutrition, dehydration, electrolyte imbalance, and aspiration pneumonia and potentially severe complications such as anastomotic fistula.^10^ The differences have been shown to be clinically and economically meaningful in that patients who develop postoperative ileus since they have longer hospital stay and higher costs compared to those who do not develop postoperative ileus.^11^, ^12^, ^13^ The pathological mechanism is multifactorial, which involved neurological, endocrine, and drug factors.^14^ Therefore, multimodal therapies are implemented in clinical practice, such as enhanced recovery after surgery (ERAS) for CRC surgery. The key components include a multidisciplinary team, multimodal methods, evidence-based approaches, and continuous audit to enhance the quality of recovery of surgical patients.^15^^,^^16^ The components of ERAS guidance need continuous development considering the current high incidence of postoperative ileus, and apart from some recommended postoperative strategies such as chewing gum, early mobilization on the day of surgery, and early intake of oral fluids and solids,^16^^,^^17^ other complementary interventions are worth exploring to tackle this challenging issue.

Acupressure is a noninvasive, simple, and user-friendly technique to enhance health, originally based on the traditional Chinese medicine theory.^18^ Different from acupuncture, which utilizes needles to stimulate acupoints, acupressure is a technique of using fingers, palms, elbows, feet, or special devices to apply pressure to different acupoints. A total of 361 main acupoints have been identified along the 14 main meridians,^19^ and each acupoint has a unique function in promoting health and preventing diseases.

### Theoretical framework underpinning acupressure

The theoretical frameworks underlying acupressure intervention are the theory of traditional Chinese medicine and the biological mechanisms of Western medicine. Traditional Chinese medicine is basically guided under the beliefs of “Unity of Universe and Human” and *yin* versus *yang,* which means opposites (ie, day vs. night, cold vs. hot, women vs. men etc.).^18^ It reveals the Chinese holistic thinking pattern of adopting a macroscopic and holistic perspective when understanding health and disease. *yin* and *yang* philosophy is the reflection of dialectical thinking pattern of finding opposition, unity, and transformation. Originating from *Huangdi Neijing*, which is believed to be the root of traditional Chinese medicine theory and practice, the Zang-fu and Meridian Theory was initially constructed through ample clinical observation and then developed over the 2000 years under the guidance of Chinese philosophy.^18^ According to the textbook, acupoint Stomach 36 (ST36 or *zusanli*) belongs to the stomach meridian and is an important acupoint to improve gastrointestinal (GI) function. Stimulating ST36 can regulate the stomach *qi* and treat stomach pain, vomiting, decreased GI function, abdominal distention, diarrhea, and constipation.^18^ Previous studies found that stimulating ST36 can significantly promote GI function after abdominal surgery.^20^^,^^21^ Acupressure at P6 (*Neiquan*) and ST36 had a significant effect at the time of the first flatus (mean, 79.97 vs. 98.09, *P* ​= ​.04) and decreased postoperative nausea and vomiting among patients with gastric cancer following a subtotal gastrectomy.^22^ Acupressure applied among patients following laparoscopic cholecystectomy operation observed a significantly shorter time to defecation (median, 29.5 vs. 46 h) compared to control group.^23^ A study applied acupressure among women undergoing planned caesarean delivery in the postoperative period, and the results showed that acupressure significantly shortened the times to first flatus (median, 1447.5 vs. 1992.5 ​min) and defecation (median, 1485 vs. 2105 min) compared to the control group.^24^

**Fig. 1:**
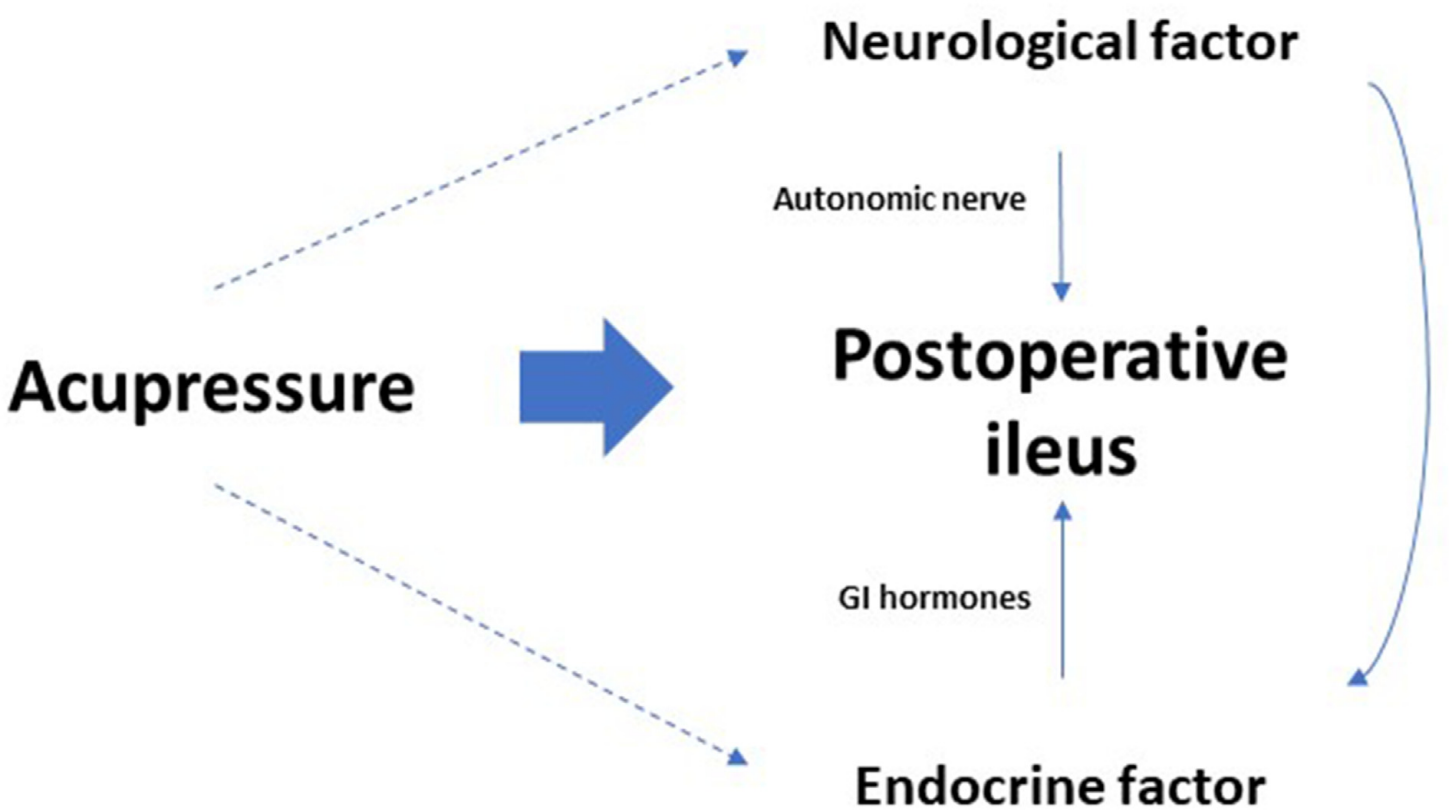
The neurological-endocrine theoretical framework and the potential impacts of acupressure.

In Western medicine, numerous basic medical experiments have been conducted to explore the biological mechanism of acupuncture. These studies can also be applied to the mechanism of acupressure. Acupuncture could alter the nervous system by changing the concentrations of K+, Na+, and Ca2+ in the neurons and the amount of neuropeptides such as endogenous opioid endorphin, leucine, encephalin, and neurotransmitters in the central nervous system.^25^^,^^26^ Acupuncture exerts local effects by releasing neuromodulators such as adenosine and systemic effects through neurotransmitters and somatoautonomic reflex.^27^ Study reported that transcutaneous electrical acupoint stimulation can induce increase in vagal activity of autonomic nervous system assessed by the heart rate variability derived from electrocardiogram.^28^ Furthermore, with the somatoautonomic reflex, electrical acupuncture affects GI hormones by increasing motilin, ghrelin, and glucagon-like peptide-1 levels and decreasing calcitonin gene-related peptide level.^28^ The neurological-endocrine theoretical framework and the potential impacts of acupressure on facilitating GI recovery are summarized in Fig. 1.

The effectiveness of acupressure on promoting postoperative GI function after CRC surgery has not been widely investigated and emphasized. Furthermore, few studies have been conducted by nurses in clinical settings on postoperative GI recovery using single acupoint pressing, which means easier and more time-saving than multiple acupoints pressing. Therefore, it is necessary to conduct the present study to investigate the effectiveness of nurse-delivered acupressure on the early postoperative GI function after CRC surgery to guide clinical practice. This study provides a safe and noninvasive means of care for the recovery of patients after CRC surgery and provides strong evidence for the promotion of acupressure in clinical settings.

## Methods

### Study design

This study is a randomized placebo-controlled trial, which was conducted in two GI surgery wards that can accommodate more than 90 inpatients in a tertiary-level comprehensive hospital affiliated to a medical university in Shandong Province, China.

#### Participants

Adult patients (≥ 18 years) who were scheduled to receive CRC surgery were eligible for the study. Inclusion criteria were determined as: (a) age older than 18 years, (b) pathologically diagnosed to be CRC, (c) able to understand the information given, (d) willing to receive acupressure, and (e) undergoing elective surgery operated on under general anesthesia. Patients with the following conditions were considered ineligible: (a) unable to communicate due to language barrier or severe cognitive impairment, (b) with skin infections or defects in lower limbs which might hinder the accuracy of locating ST36 or get infection worse, (c) scheduled to have more than one surgery during the hospitalization, and (d) with coagulation disorders or peripheral vascular disease or having signs of compromised circulation in lower limbs.

#### Randomization and masking

A sequence of block randomized number with a block size of eight generated by a website (www.randomisation.com), as seen in Table 1, was placed into sequentially numbered, opaque, and sealed envelopes by a research nurse who did not participate in this study.^29^ The researcher (first author) opened these envelopes one day before surgery, according to the sequence of the participants’ surgery. The patients and medical staff (except one research nurse) were unaware of group allocation.

**Table 1:** Randomization plan of the current study.

Block	1								2							
Patient ID	1	2	3	4	5	6	7	8	9	10	11	12	13	14	15	16
Generated number	2	1	7	5	8	4	6	3	7	6	3	5	4	2	8	1
Group	1	2	2	2	1	1	1	2	2	1	2	2	1	1	1	2

Group 1 represents intervention group while Group 2 represents control group.

#### Sample size

Sample size was calculated based on previous similar studies (in terms of intervention, population, and primary outcome), which were published before the beginning of this study. Chao et al.’s study (2013) with an effect size of 0.797 on the primary outcome of time to first flatus passage was identified as the latest study similar to the present study. However, since Chao et al.’s study (2013) was rated as high risk of bias by the Effective Public Health Practice Project, we conservatively estimated a smaller effect size as 0.55. Sample size was calculated by mean difference of time to have first flatus passage by two-tailed *t*-test with the alpha at .05 and the power at .80 using G∗power (version 3.1), the final sample size was calculated at 106.

**Fig. 2:**
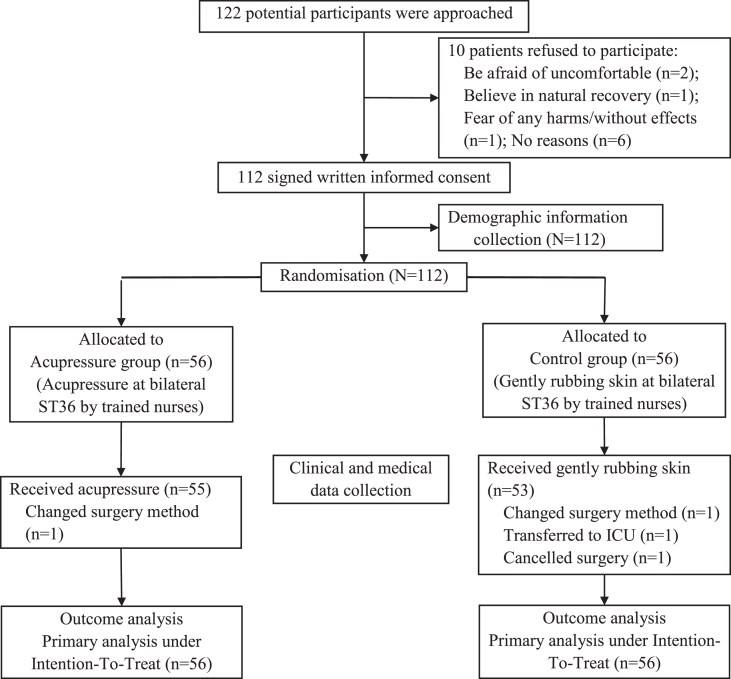
Flowchart of subject recruitment, group allocation and data collection.

In this study, a total of 122 potentially eligible participants were approached and 112 were recruited. After randomization and demographic information collection, four patients were further excluded with reasons shown in Fig. 2. Under the intention-to-treat principle, 56 patients in each group were analyzed. The recruitment process and group allocation are also displayed in Fig. 2.

### Study procedures

The procedure of acupressure was developed based on findings of our previous systematic review,^30^ suggestions from textbooks and a physician (GD). Two research nurses who are similar in demographic characteristics (age, nursing qualification and duration of nursing education) conducted acupressure for patients.

#### Qualification training

Both nurses have studied traditional Chinese medicine course in the university. Furthermore, prior to the study, they received a training session on locating ST36 and the technique of using an acupen by a licensed physician of traditional Chinese medicine (GD). During the training session, the research nurses repeatedly practiced acupressure procedure and force application using an acupen until they learn the technique. The physician monitored the practices and determined the qualification of the research nurses. Furthermore, the research nurses’ competency in conducting acupressure on participants was supervised by the physician during the pilot study. During the study, the same nurse was responsible for all treatment sessions of the same patient.

#### Acupressure procedure

**Fig. 3:**
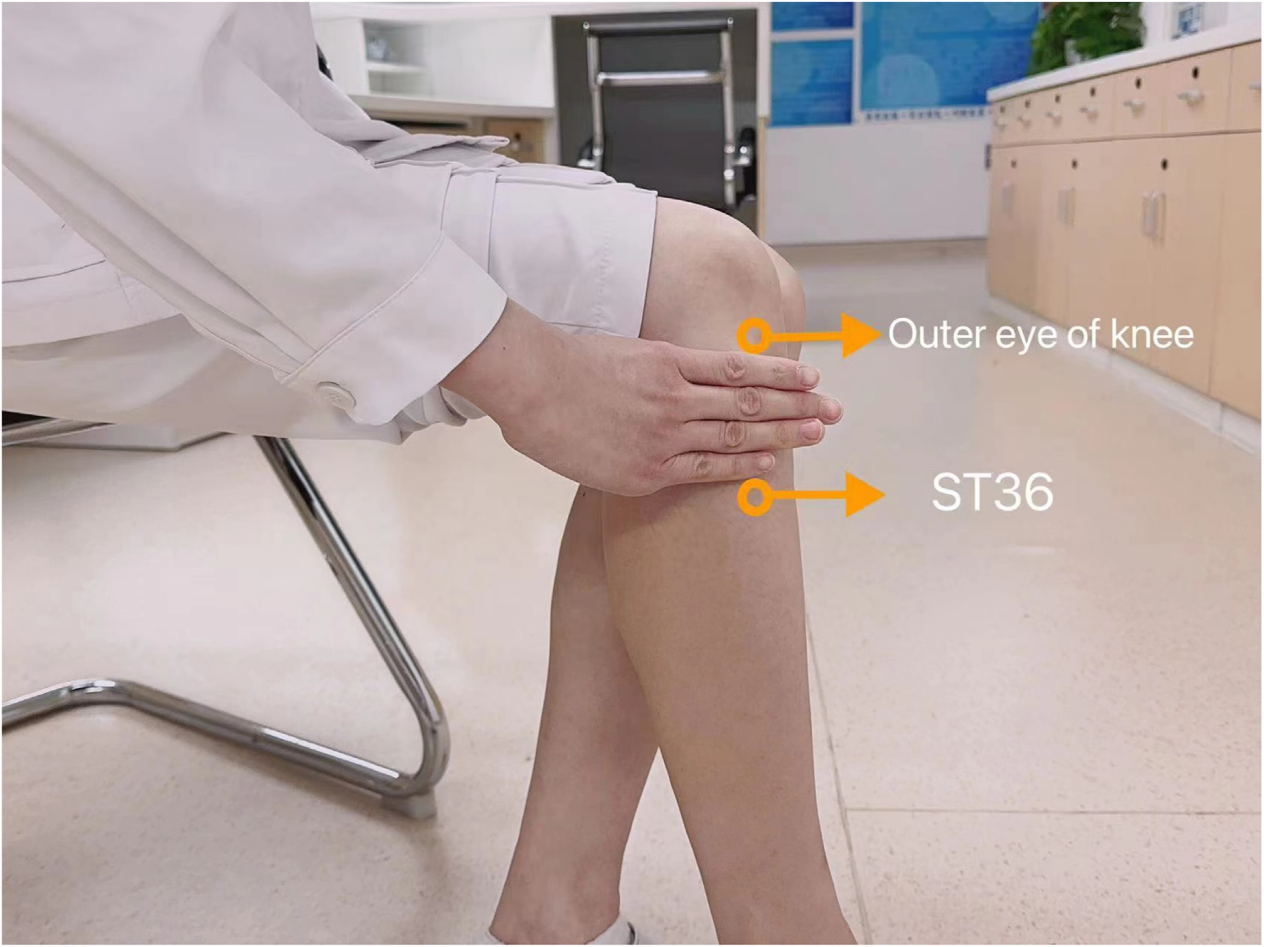
Location of ST36 (Stomach 36 or *Zusanli*). Note. ST36 is located at lateral to the tibia and three *cun* (equal to the width of the patient's four fingers at the level of proximal interphalangeal joint of the index finger) down from the patella. Photo courtesy of Xue Zhang (used with permission).

The nurses located and marked the acupoint ST36, which is located at lateral to the tibia and three *cun* (equal to the width of the patient's four fingers at the level of proximal interphalangeal joint of the index finger) down from the patella, as shown in Fig. 3.^31^ The acupressure was practiced at bilateral ST36 by a nonelectronic metal acupen, which is used for pressing acupoints instead of using fingers. The acupressure process was repeated by pressing once per second and lasted for 5 ​min on each side.^32^^,^^33^ The frequency was twice a day starting from day 1 (during 8:00–10:00 am and 3:00–5:00 pm) and lasted for 5 days after operation.^32^ The pressing force of acupressure was approximately 2–3 kg but with adjustments according to patients' feedback based on the individualized principle.^34^ During the process, vital signs (heart rate and blood pressure level on bedside monitors, if available) and patients' reports of discomfort were carefully monitored to ensure patients' safety.

Patients in the control group received gently rubbing the skin on ST36 with no pressure. The acupressure procedure, frequency, and duration were the same as those with the intervention group. All other medical and nursing care in both groups followed the usual care in the hospital.

#### Outcome measures

Demographic information was collected before randomization. Medical information was retrieved from medical records by the researcher. The primary outcome for GI function had routinely adopted the first passage of flatus and defecation,^35^ which means the time interval (hours) from the end of surgery to the first passage of flatus and defecation. The secondary outcomes involved the degree of abdominal distention and bowel motility. The degree of abdominal distention was evaluated by the 11-scale Numerical Rating Scale (NRS) (0–10) (0 ​= ​no complaints at all; 10 ​= ​the most severe symptoms).^36^ NRS is sensitive and simple to measure patients’ experiences and is both reliable and valid, thus is widely used in clinical environment.^37^^,^^38^ Bowel motility was evaluated by a stethoscope to assess the number of intestinal peristalsis sounds in 1 ​min on the body surface above the ileocecal valve in the lower right corner of the abdomen.^39^ These outcomes were evaluated before the first and after the last acupressure each day in both groups, while the highest degree/number each day was utilized for outcome analyses. The definitions and measurement of other variables in this study are shown in Appendix A. The intention-to-treat principle was adopted.^40^

### Data analysis

The statistical package SPSS (version 25.0) was used for data analysis. Descriptive statistics including mean ​± ​standard deviation (SD) and median and interquartile range (IQR) were displayed for continuous variables, whereas frequencies and percentage were presented for categorical variables. Comparisons of demographic and clinical characteristics between the intervention and control groups were conducted using the *t*-test and Mann–Whitney U test for continuous data based on the normality test results. Chi-square test or Fisher's exact test was employed for categorical data. The differences in time to have first passage of flatus and defecation between groups were compared by independent *t*-test and regression analyses with covariates as explanatory variables adjusted.^41^ The covariates were determined on the basis of the univariate analysis results (variables with *P* ​< ​0.15)^42^ and literature, which showed the risk factors of the primary outcomes. Mann–Whitney U test or *t*-test was used to compare the differences of area under the curve (AUC) for repeated measures of abdominal distention and bowel motility between groups and subgroups. The AUC is the outcome of integration over some range of values or a summation of polygons within consecutive intervals.^43^ The AUC with respect to the ground, that is the area between the curve (trajectory for longitudinal data) and the x-axis (absence of behavior), was calculated in this study. For all outcome analyses in this study, two-tailed *P* ​< ​0.05 was considered as statistically significant.

### Ethical considerations

This study was conducted after obtaining approval from the Joint Chinese University of Hong Kong-New Territories East Cluster Clinical Research Ethics Committee (IRB No. 2017.363-T) and the participating hospital (IRB No. KYLL-2017-585). Furthermore, this study has registered in the Chinese Clinical Trial Registry (ChiCTR-IOR-17012460) and Chinese University of Hong Kong (CUHK) Centre for Clinical Research and Biostatistics (CUHK_CCRB00564). Registration occurred before the first participant was recruited. All participants provided written informed consent. The study followed the Consolidated Standards of Reporting Trials.^44^

## Results

There were 51.8% male patients, and the mean ages for the intervention and control groups were 58.89 and 58.43 years (*P* ​> ​0.05), respectively. The average doses of acupressure received in the intervention and control groups were 8.89 ​± ​2.07 versus 8.50 ​± ​1.82 out of 10 doses in total (*P* ​> ​0.05), respectively. Other demographic and clinical characteristics between the intervention and control groups were also comparable, as shown in Table 2.

**Table 2:** Comparisons of demographic and clinical characteristics between intervention and control groups.

Variables	Intervention group (*n* ​= ​56)	Control group (*n* ​= ​56)	*P*
Gender			
Male	29 (51.8%)	29 (51.8%)	1.00
Female	27 (48.2%)	27 (48.2%)	
Age, Mean ± SD, years	58.89 ​± ​13.37	58.43 ​± ​11.94	0.85
Range	29–83	31–83	
BMI (kg/m^2^)	24.52 ​± ​2.79	24.93 ​± ​3.61	0.52
Range	20.06–31.59	18.83–32.85	
Education level^a^			
Primary school or below	21 (37.5%)	20 (37.7%)	0.55
Secondary school	21 (37.5%)	14 (26.4%)	
High school	9 (16.1%)	12 (22.6%)	
University or above	5 (8.9%)	7 (13.2%)	
Previous smoking	19 (33.9%)	15 (26.8%)	0.41
Previous abdominal surgery	20 (35.7%)	12 (21.4%)	0.09
Radiotherapy before surgery	1 (1.8%)	1 (1.8%)	1.00
Chemotherapy before surgery	7 (12.5%)	4 (7.1%)	0.34
Comorbidity			
Hypertension	20 (35.7%)	15 (26.8%)	0.31
Diabetes	4 (7.1%)	8 (14.3%)	0.22
Cardiovascular disease	13 (23.2%)	10 (17.9%)	0.48
Cerebrovascular disease	4 (7.1%)	2 (3.6%)	0.34
Decreased lung function	4 (7.1%)	3 (5.4%)	0.70
Anesthesia^a^			
General anesthesia	37 (67.3%)	42 (76.4%)	0.29
Combined anesthesia	18 (32.7%)	13 (23.6%)	
Laparoscopic surgery	43 (78.2%)	49 (90.7%)	0.07
Location of cancer			
Colon	20 (35.7%)	20 (35.7%)	1.00
Rectum	36 (64.3%)	36 (64.3%)	
Duration of surgery (min)	193.66 ​± ​66.40	182.02 ​± ​62.34	0.35
Duration of anesthesia (min)	244.82 ​± ​71.40	234.42 ​± ​69.88	0.45
The time interval of the first instance out of bed (h)	58.91 ​± ​19.50	52.80 ​± ​21.68	0.13
Liquid food intake interval (h)	79.69 ​± ​26.20	75.78 ​± ​27.92	0.45
Water intake time interval (h)^a^			0.06
≤ 24	32 (59.3%)	42 (80.8%)	
25–48	13 (24.1%)	7 (13.5%)	
49–72	2 (3.7%)	2 (3.8%)	
> 72	7 (13.0%)	1 (1.9%)	
Duration of nasogastric tube (h)^a^			0.06
< 24	33 (60.0%)	42 (79.2%)	
24–47	12 (21.8%)	8 (15.1%)	
≥ 48	10 (18.2%)	3 (5.7%)	
Doses of acupressure	8.89 ​± ​2.07	8.50 ​± ​1.82	0.30
Complications			
Yes	7 (12.5%)	7 (12.5%)	1.00
No	49 (87.5%)	49 (87.5%)	
Length of hospital stay (days)	19.23 ​± ​7.10	17.71 ​± ​7.17	0.27

BMI, body mass index.

a Having missing data, thus the number of patients for categorical variables was not necessarily equal to the total number of each group according to the intention-to-treat principle.

### Effects of acupressure on the flatus and defecation passage

The differences of time to have the first passage of flatus and defecation were not statistically significant between groups (*P* ​= ​0.15 and 0.62 for flatus and defecation passage, respectively) using the univariate Student's *t*-test.

As displayed in Table 3, after variables of age, previous abdominal surgery, laparoscopic surgery, anesthesia duration, nasogastric tube duration, water intake time interval, and the time interval of the first instance out of bed were adjusted, acupressure could significantly shorten the time interval of first flatus passage by 11.08 ​h (95%CI: −19.36 to −2.81; *P* ​< ​0.01). However, it did not significantly influence the first passage of defecation (*β* ​= ​−5.90, 95%CI: −19.34 to 7.55; *P* ​= ​0.39).

**Table 3:** Multivariate linear regression of the time of first flatus and defecation passage.

Variables	Intervention group (M±SD) (h)	Control group (M±SD) (h)	MD (h)	Coefficient *β*	*β* 95% CI	*P*
The first passage of flatus	44.39 ​± ​22.37	50.16 ​± ​20.11	−5.77	−11.08	−19.36 to −2.81	0.01^a^
The first passage of defecation	77.00 ​± ​36.27	80.08 ​± ​28.88	−3.08	−5.90	−19.34 to 7.55	0.39

CI, confidence interval; M, mean; SD, standard deviation; MD, mean difference, the values in the intervention group minus those in the control group.

a *P* ​< ​.05; adjusted variables include age, previous abdominal surgery, laparoscopic surgery, anesthesia duration, nasogastric tube duration, water intake time interval, and the time interval of the first instance out of bed.

### Effects of acupressure on postoperative abdominal distention and bowel motility

The average degrees of abdominal distention were 1.33 ​± ​2.02 versus 1.68 ​± ​1.77 (median, 0.00 vs. 1.00) in the intervention and control groups, respectively. Additionally, bowel motility was 3.04 ​± ​1.97 versus 2.69 ​± ​1.38 (median, 3.00 vs. 3.00) bowel sounds per minute in the intervention and control groups, respectively.

A total of 560 observations for each repeated measured outcome were recorded, with the percentage of total missing data at 3.75%. The demographic and clinical characteristics of the patients between with and without missing data group were comparable. The AUC for abdominal distention was 5.68 ​± ​5.24 and 5.92 ​± ​4.03 in the intervention and control groups (*P* ​= ​0.35), while the AUC for bowel motility was 12.09 ​± ​4.70 and 11.51 ​± ​3.00 (*P* ​= ​0.45). When analyzed in two subgroups of patients with < 60 and ​≥ ​60 years old separately, the subgroup of patients ≥ 60 years old had better average improvements in both outcomes although the differences were not statistically significant (*P* ​> ​0.05). The description and comparisons of abdominal distention and bowel motility in the intervention and control groups are displayed in Table 4.

**Table 4:** Description and comparisons of abdominal distention and bowel motility in intervention and control groups within 5 days postoperation.

Outcome variables (time point)	Intervention group (*n* ​= ​56)			Control group (*n* ​= ​56)			*P*
	Mean	Median	SD	Mean	Median	SD	
Abdominal distention							
D1	1.16	0.00	1.51	1.75	1.00	1.95	
D2	1.46	0.00	2.05	2.09	2.00	1.68	
D3	1.71	1.00	2.08	1.92	2.00	1.77	
D4	1.34	0.00	2.01	1.60	1.00	1.96	
D5	0.98	0.00	1.67	0.98	1.00	1.23	
Total	1.33	0.00	2.02	1.68	1.00	1.77	0.035^a^
AUC	5.68	5.00	5.24	5.92	6.00	4.03	0.35
60 years	6.26	5.00	6.00	6.02	6.25	3.40	0.85
≥60 years	5.14	4.00	4.46	6.02	4.75	4.69	0.49
Bowel motility							
D1	2.02	2.00	1.80	1.21	1.00	1.23	
D2	2.86	2.00	1.99	2.67	3.00	1.40	
D3	3.11	3.00	1.83	3.08	3.00	1.23	
D4	3.32	3.00	1.77	3.16	3.00	0.84	
D5	4.00	4.00	1.96	3.37	3.00	0.91	
Total	3.04	3.00	1.97	2.69	3.00	1.38	0.017^a^
AUC	12.09	12.00	4.70	11.51	11.50	3.00	0.45
< 60 years	12.35	12.00	5.00	12.30	12.00	2.81	0.96
≥ 60 years	11.84	12.25	4.67	10.64	11.25	3.08	0.29

Abbreviations: AUC, area under the curve; D, postoperative day; SD, standard deviation.

a *P* ​< ​.05.

## Discussion

The present study revealed that nurse-delivered acupressure at ST36 has positive influences on early postoperative GI function among patients undergoing CRC surgery. This study filled the gap in literature, and the procedure may be reproducible and applied to other studies or clinical practices.

### Effects of acupressure on the flatus and defecation passage

The average time to have first flatus passage was 6 ​h shorter in the intervention group than the control group. The absolute improvement was small, and the univariate analysis did not identify statistical significance. The reasons might be related to baseline characteristics of the participants. Unfavorable characteristics for GI function including having previous abdominal surgery,^45^ open surgery type,^46^ and long duration (> 24 ​h) of nasogastric tube placement^47^ appeared more prevalent in the intervention group, and more patients were older than 60 years (52.6% vs. 48.1%), who generally have decreased bowel function, in the intervention group, although all the differences were not significant between the groups. That means, despite of the disadvantages in the baseline characteristics, acupressure still shortened the average absolute time to have the first flatus passage by 6 ​h and this may have clinical significance considering that earlier recovery of GI function means earlier relief of abdominal dysfunctional symptoms. With the adjustment of these potential influencing factors in the multivariate regression analysis, the benefits of acupressure were enhanced to significantly shortening the time of first flatus passage by 11.08 ​h. This result was similar to previous studies. A study of acupressure at ST36 among patients with CRC with surgery reveals that acupressure significantly shortened the time to have first flatus passage by 19.92 ​h compared with the control group.^31^ Another study of acupressure at two acupoints, P6 (*Neiguan*, located on the volar side of the forearm, two inches above the wrist stripe, between the palmaris longus tendon and the radial flexor tendon) and ST36 indicates that acupressure significantly shortened the time to have first flatus passage by 18 ​h compared with the usual care group among patients undergoing gastric cancer surgery.^22^ As introduced in the theoretical framework underpinning acupressure, acupressure promoted GI movement by systemic effects through neurotransmitters and somatoautonomic reflex and GI hormones.^27^^,^^28^ Although the biological mechanisms of acupuncture and acupressure have been actively investigated, their clinical effects cannot be fully explained through a single mechanism such as neurobiological channels or endocrine factors. Therefore, the modern interpretation of acupressure is not definitive, and the mechanisms of Western medicine and traditional Chinese medicine are complementary rather than exclusive.

It is noteworthy that the time to have flatus passage in the current study was shorter than previous similar studies. In the study of Chao et al (2013),^31^ the time of first postoperative flatus passage was 66.72 and 86.64 ​h in the intervention and control groups, respectively. The difference is probably due to the fact that all participants in Chao et al.’s study underwent open abdominal colectomy or rectal resection, whereas the majority of patients (82.1%) in the present study underwent laparoscopic surgery for CRC. Another study shows that the time of first flatus passage was 80 ​h in the acupressure group and 98 ​h in the usual care group.^22^ The prolonged duration to have first flatus passage might be because only three sessions of acupressure were delivered over the first 3 days after operation compared with 10 sessions of acupressure over the first 5 days after operation in the current study. The dose–response association between acupressure and postoperative GI symptom relief was suggested in the systematic review conducted by our research team.^30^

The routine clinical practices can have a great impact on the GI recovery of patients. In this study, 40.0% of the patients kept the nasogastric tubes for more than 24 ​h in the intervention group and it was marginally significant longer duration of nasogastric tubes than the control group. Increased duration of nasogastric tube placement can result in decreased bowel motility.^47^ This relationship may be explained partly by the delayed oral feeding of water due to the placement of nasogastric tubes. A Cochrane systematic review demonstrates that early oral feeding of fluids or food within 24 ​h after abdominal gynecologic surgery is associated with shorter time to the presence of bowel sounds by 0.32 days (*P* ​= ​0.03) than delayed feeding after 24 ​h.^48^

Acupressure was found to have no significant influences on the time of first defecation passage both in Student's *t*-test and multivariate regression analysis. This finding is consistent with previous studies.^22^^,^^31^^,^^49^ One possible explanation is that patients take different amounts and types of food after flatus passage, which can influence the time to have defecation passage. Future studies may measure the amount and types of food intake to examine this factor.

### Effects of acupressure on postoperative abdominal distention and bowel motility

Abdominal distention, which is a typical symptom of postoperative ileus, is a common complaint among patients after CRC surgery and can increase the risks of anastomotic fistula, prolong oral food intake time and cause abdominal pain, thus requiring treatments.^13^ As a result of decreased bowel motility after CRC operation, gas and fluid accumulate in the GI, which leads to dilated GI and abdominal distention. The early intake of oral food may also lead to abdominal distention given the not fully recovered GI movement. We observed several cases of withholding oral food intake due to severe abdominal distention, and some of them might need reinsertion of nasogastric tubes to decompress the GI tract. In our clinical practice, severe abdominal distention may be treated with reinsertion of nasogastric tubes, drinking paraffin oil, and usage of glycerine enema; most patients were not treated and only waited for the symptom to be automatically alleviated. A previous similar study indicates that, compared to usual care, acupressure can significantly reduce the intensity of abdominal distention from “very severe” or “severe” to “moderate” and “slight” among patients undergoing laparoscopic abdominal surgery on the first postoperative day.^50^ In the future study, abdominal X-ray may be more sensitive to diagnose bowel distention with air fluid levels at various points in the intestine than self-reported distention degree by NRS.^13^

In subgroup analysis of patients based on age, we found that acupressure improved bowel motility even more among elderly patients by about one bowel sound per minute. Acupressure at ST36 in a previous study also reveals significantly increased abdominal motility by approximately one bowel sound per minute compared with the control group during the first 2 days after surgery.^31^ Based on the findings of this study, implementing acupressure at ST36 in clinical settings will be valuable when reduced bowel motility is the main symptom concern among patients who have undergone surgery for CRC especially for the elderly.

### Limitations

This study had limitations that need explanations. First, this is a single-center study, which may compromise the external validity of the study. Second, owing to the multifactorial origin, future subgroup analyses based on surgery type, location, and so on, are encouraged to minimize the variations. Third, the self-reported abdominal distention severity was relatively subjective and time-dependent, which may bring risks of bias. Additionally, during the study, we did not measure the exact ERAS adherence percentage of the research hospital, which may influence the assurance of the effect size of acupressure. Finally, we did not conduct follow-ups of participants after their discharge and examine the long-term effect of acupressure on GI function. Future studies need to address the limitations mentioned above and a multicenter design with a larger sample size should be adopted. A longitudinal study with follow-ups is also essential to evaluate the long-term effects and complications.

### Implications for practice

The present study demonstrated that acupressure is a promising technique to be applied in the hospital setting after evaluating various stakeholders’ opinions. Nurse-delivered acupressure is noninvasive and effective to improve early postoperative GI function after CRC surgery. Nurse-delivered acupressure could be a new solution and incorporated into ERAS protocol for CRC surgery. Technologies, such as abdominal X-ray, can be applied to provide objective data on GI recovery. Future qualitative studies are also essential to explore experiences and perceptions of stakeholders (ie, patients, nurses, doctors and managers, etc.).

## Conclusions

This study provided evidence that nurse-delivered acupressure had a positive influence on early postoperative GI function after CRC surgery. Hospitals may consider to incorporate nurse-delivered acupressure intervention in its clinical practice to better promote early recovery of patients.

## Figures and Tables

**Table:** Definitions and measurements of variables in this study

Variables	Definitions	Measurements
Time to have the first flatus passage	The time between the end of surgery and the first passage of flatus.	Clinical nurses directly asked patients the exact time to have the first flatus passage after surgery every day and recorded them in nursing records.
Time to have the first defecation passage	The time between the end of surgery and the first passage of defecation.	Clinical nurses directly asked patients the exact time to have the first defecation passage after surgery every day and recorded them in nursing records.
Bowel motility	Intestinal peristalsis, a form of radially symmetric contraction and relaxation of muscles that propagate down the tube and along the anterograde direction.	The outcome assessors assessed the patients' bowel motility on abdominal auscultation by using a stethoscope for one full minute on the body surface above the ileocecal valve in the lower right corner of the abdomen before the first and after the last acupressure or sham acupressure each day (twice a day) until postoperative day 5.
Degree of abdominal distention	Gas and fluid accumulate in the GI, which leads to dilated GI and abdominal distention.	The outcome assessors directly asked patients perceived degree of abdominal distention by NRS from 0 to 10 (0 represents no distention at all and 10 represents the most severe distention) before the first and after the last acupressure each day (twice a day) until postoperative day 5.
Dose of acupressure	The number of times patients received acupressure (the total designed acupressure was 10 times).	Once patients received acupressure, the duration, outcomes and side effects were recorded in the acupressure logbook by the researchers.
Water intake time interval	The time between the end of surgery and the first drinking small amount of water.	The time was retrieved from medical records.
Liquid food intake interval	The time between the end of surgery and the first drinking rice soup based on the routine recommendation in the research hospital.	The time was retrieved from medical records.
The time interval of the first instance out of bed	The time between the end of surgery and first leaving bed, including standing or walking beside bed or going to toilet.	The outcome assessors directly asked patients the exact time to leave bed every day.
Duration of nasogastric tube placement	The time between the placement and removal of a nasogastric tube.	The outcome assessors directly asked patients the exact time to have their nasogastric tube placed and removed.

**Table:** Clinical practice in the research hospital

Recommendations from enhanced recovery after surgery (ERAS)	Clinical practice in the research hospital
***Preoperative***	
Early identification and optimization of medical comorbidities	Full
Smoking cessation	Full
Improved nutritional status	Full
Prehabilitation for ideal physical fitness	Partly
Preadmission education including expectations for postoperative care and recovery	Partly
Reduce preoperative anxiety of patient and family	Partly
Prepare patient with realistic expectations	Partly
Adherence to current practice guidelines for fasting (minimization of prolonged fasting)	Not
Clear fluid and carbohydrate-rich drink loading 2 ​h before surgery	Not
Omission of selective bowel preparation (no routine bowel cleansing procedures)	Not
Appropriate antibiotic prophylaxis	Full
Appropriate thromboprophylaxis	Partly
***Intraoperative***	
Use of short-acting anesthetic agents	Full
Support rapid emergence and recovery	Full
Opioid-sparing pain management	Full
Multimodal techniques to include pre-emptive regional anesthesia/analgesia	Not
Avoidance of postoperative drains and nasogastric tubes help minimize discomfort and support early mobilization	Not
Judicious management of intraoperative fluid replacement	Partly
Maintenance of normothermia (body warmer/warm intravenous fluids)	Partly
***Postoperative***	
Implementation of multimodal opioid-sparing analgesia to avoid delayed gut function	Partly
Midthoracic epidural anesthesia/analgesia	Not
Prevention of nausea and vomiting	Partly
Early oral nutrition as tolerated	Partly
Stimulation of gut motility	Partly
Restricted amounts of intravenous fluids	Partly
Early mobilization	Partly
Early removal of urinary catheter	Full

## Data Availability

The data that support the findings of this study are available from the corresponding author, upon reasonable request.

## Credit author statement

**Yunhong Liu**: Conceptualization, Methodology, Data curation, Writing – original draft. **Carmen WH Chan**: Supervision, Methodology, Validation, Writing – review and editing. **Ka Ming Chow**: Supervision, Validation, Writing – review and editing. **Binbin Zhang**: Project administration. **Xue Zhang**: Investigation. **Chao Wang**: Resources. **Guangzhong Du**: Methodology. All authors had full access to all data in the study, and the corresponding author had fifinal responsibility for the decision to submit for publication. The corresponding author attests that all listed authors meet authorship criteria and that no others meeting the criteria have been omitted.
